# Causes of recurrence in laparoscopic inguinal hernia repair

**DOI:** 10.4103/0972-9941.27736

**Published:** 2006-09

**Authors:** Jan F Kukleta

**Affiliations:** Klinik Im Park, Zurich, Switzerland

**Keywords:** Endoscopic hernia repair, inguinal hernia, recurrence, trans-abdominal preperitoneal and total extraperitoneal

## Abstract

**Aim:**

The analysis of possible mechanisms of repair failure is a necessary instrument and the best way to decrease the recurrence rate and improve the overall results. Avoiding historical errors and learning from the reported pitfalls and mistakes helps to standardize the relatively new laparoscopic techniques of trans-abdominal preperitoneal and total extraperitoneal.

**Materials and Methods:**

The video tapes of all primary laparoscopic repairs done by the author that led to recurrence were retrospectively analyzed and compared with findings at the second laparoscopic repair. A review of the available cases of recurrences occurring between 1994 and 2003 is the basis of this report.

**Summary:**

Adequate mesh size, porosity of mesh material, slitting of the mesh, correct and generous dissection of preperitoneal space and wrinkle-free placement of the mesh seem to be the more important factors in avoiding recurrence rather than strength of the material or strong penetrating fixation. Special attention should be paid to preperitoneal lipoma as a possible overlooked herniation or potential future pseudorecurrence despite nondislocated correctly positioned mesh.

**Conclusion:**

Laparoscopic hernia repair is a complex but very efficient method in experienced hands. To achieve the best possible results, it requires an acceptance of a longer learning curve, structured well-mentored training and high level of standardization of the operative procedure.

Inguinal hernia repair is one of the most frequently performed elective operations today. For many years, recurrence was the only criterion by which the quality of a hernia repair was measured. Recurrence rates of over 15% for primary repair were accepted before the mesh techniques were introduced. Following growing acceptance of nonabsorbable implants and their wider use, both in open and endoscopic repairs, dramatic reduction of recurrence rates has been demonstrated. Chronic pain and infection, as aspects of impaired quality of life and increased socioeconomic cost, gained the necessary attention due to social consequences, which seem to be even more important than the recurrence itself.

The advent of laparoscopic hernia repair has brought about not only a different approach but in almost all cases, implantation of a nonabsorbable mesh. Additionally, it has resulted in an unpleasant reality of a long learning curve of laparoscopic technique and an unexpected complexity of repair of a frequent pathology, which until then was considered trivial and simple when repaired by sutures.

Despite the marked improvements, admittedly, even a ‘low’ recurrence rate is a failure of achieving the primary goal of any repair. The recognition of the causes of recurrence makes their prevention / elimination possible. The analysis of the possible reasons may help us to reduce the recurrence rate *per se;* to sharpen the indications for repairs; to improve recognition of the risk factors; to develop, learn and apply the best practice, based on clinical evidence. First then, we should start discussing the cost-utility issues.

## MATERIALS AND METHODS

The analysis of possible causes of recurrence was based on individual reports, retrospective studies, comparative studies, reviewing of videos and reoperations. Since the introduction of laparoscopic hernia repair techniques in the early ‘90s, there has been an understandable effort to analyze the procedures, the mesh materials used, mesh size, its fixation and other probable circumstances that may lead to a recurrence[1–4] [Table 1].

**Table 1:** Possible causes of recurrence in laparoscopic inguinal hernia repair

Technique	Lack of experience
	Insufficient extent of dissection
	Missed hernia
	Preperitoneal lipoma
	Suboptimal mesh placement
	Inappropriate retention/fixation
	Mesh lifted by hematoma
	Inferior lateral mesh edge lifted at closure
Material	Micro-porous mesh
	Heavyweight mesh/ excessive shrinkage
	Size too small
	Insufficient overlap in relation to shrinkage
	Mesh slit
	Mesh protrusion
Risk factors	Collagen disease
	Smoking
	Obesity
	Malnutrition
	Diabetes Type II
	Chronic lung disease
	Coagulopathy
	Steroids
	Radiotherapy, chemotherapy
	Jaundice
	Male gender
	Anemia

All laparoscopic hernia repairs done by the author since 1992 (more than 2,500) have been videotaped or digitally recorded. The recordings of all operations that resulted in a recurrence were retrospectively analyzed and compared with the intraoperative findings of the patients who were reoperated laparoscopically.

The available literature concerning the issue of recurrence after laparoscopic repair was reviewed. By gaining knowledge through own experience and correcting the obvious technical errors reported by others, we have laid the foundations for standardization of the technique of laparoscopic hernia repair.

## DISCUSSION

Hypothetically a nonabsorbable mesh of adequate chemical and physical properties, adequate size and adequate overlap, well adapted to the underlying tissue and enabling a good connective tissue ingrowth and one that does not dislocate should prevent recurrence if it withstands the local pressure forces that caused the hernia to occur.

What then can cause mesh dislocation or failure? The factors involved are insufficient size, wrong/defective material, incorrect placement, immediate or very early displacement by folding, lifting by a hematoma or urinary retention, late displacement by insufficient scar tissue ingrowth, mesh protrusion, collagen disease or pronounced shrinkage. Despite the correct and stable mesh position, there is still a limited risk of a late sliding of the retroperitoneal fat under/ in front of the mesh into the enlarged inner ring.

Until 1995 the recognized reasons of recurrence were lack of surgical experience, inadequate mesh size and its fixation and overlooked or missed hernias. The reported recurrence rate was lower with a large well-anchored mesh;[1] and among 19 recurrences, in 60% the mesh was too small,[2] in 30% the fixation was found to be insufficient and in 20% the hernia was never repaired. Technical factors were found responsible for nearly all recurrences.

**Figure 1:**
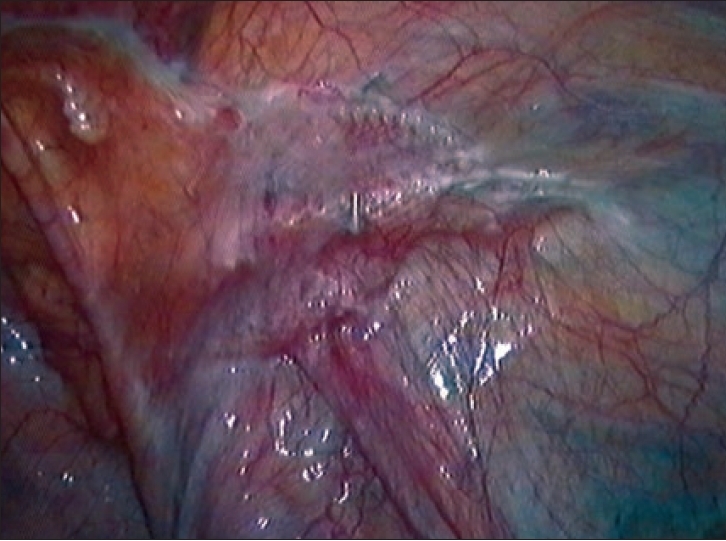
No clinical recurrence but insufficient resulting coverage of myopectineal orifice due to small size, folds, wrinkles and shrinkage

In a multicenter study[5] published in 1998, additional technical errors were identified: missed cord lipomas[5,6] and herniation through the keyhole (mesh slit),[7,8] inadequate dissection, insufficient overlapping of the myopectineal orifice, folding or twisting of the mesh and dislocation due to a hematoma[7] [Figure 1].

The ongoing discussion about the usefulness / necessity of the slit in the mesh was well responded by Leibl *et al* in 2000.[8] Avoiding slitting of the mesh and increasing its size reduced their recurrence rate from 2.8 to 0.36%. This requires more generous dissection of preperitoneal space but eliminates potential herniation through the slit or strangulation of the cord structures completely and reduces the risk of genitofemoral neuropathy.

### Mesh size

To completely cover and sufficiently overlap the entire myopectineal orifice, the size of the implanted material gradually increased in course of time.[4,8,9,10] The established size in 2006 is 15 cm × 10 cm per unilateral hernia, with minor deviations [Figure 1].

### Mesh material

The mechanical strength of available meshes exceeds the intra-abdominal peak pressures and by far even the lightweight meshes are strong enough for inguinal repair. Different mesh constructions demonstrate a variable extent of protrusion. This fact should be respected, especially in big direct hernias, where the resulting bulge - a type of pseudohernia - may become symptomatic.[11]

**Figure 2:**
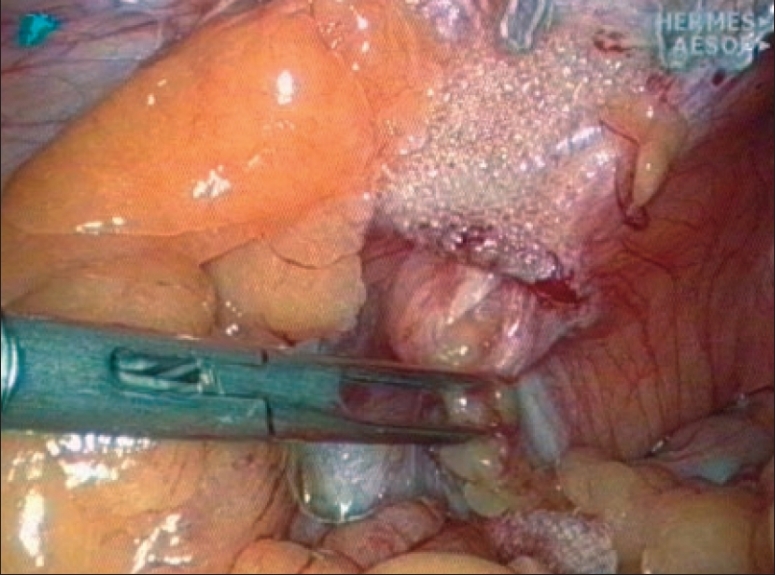
Pronounced shrinkage of a heavyweight mesh permitting a multi-orifice recurrence

**Figure 3:**
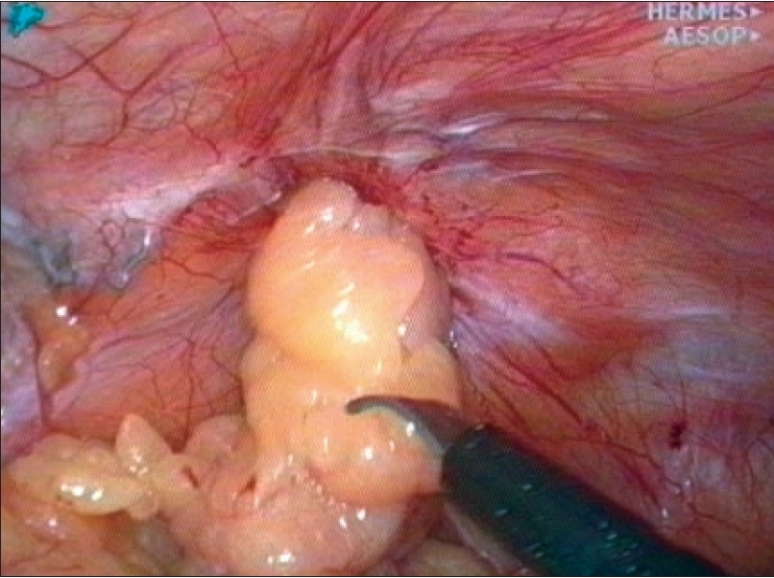
Stapling doesn't prevent dislocation of mesh caused by shrinkage

An important contribution to the understanding of interaction of the living tissue with the implanted mesh material is being made since years by the ‘Aachen group.’ The negative impact of pronounced shrinkage of the traditional heavyweight meshes was recognized as an important factor promoting recurrence [Figures 2 and 3]. Schumpelick and coauthors have induced and facilitated the logical trend of the use of lightweight meshes.[12,13] The new macroporous compound meshes present both the successful reduction of the overall foreign body amount and the preservation of mesh elasticity after the scar tissue ingrowth, due to very limited shrinkage and reduced bridging effect.[12,14]

### Fixation of the mesh [Figure 4]

**Figure 4:**
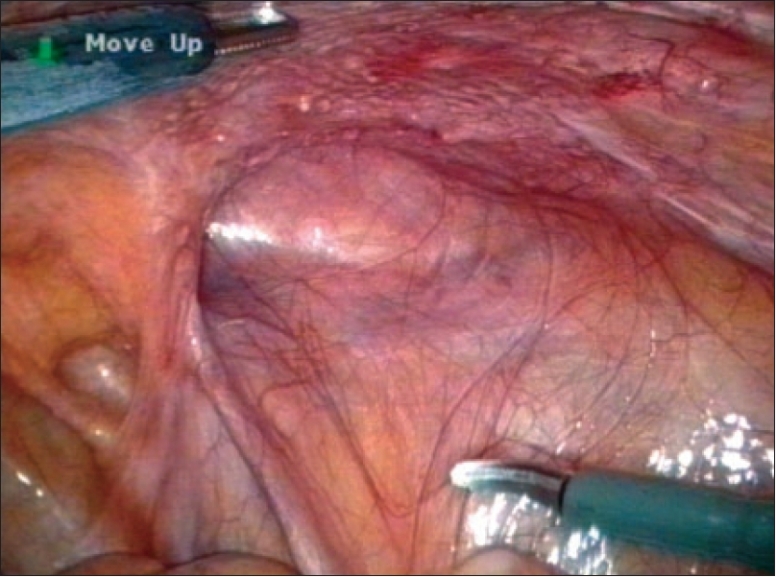
Complete dislocation of intraoperatively correctly placed and stapled lightweight mesh

In the early years of laparoscopic hernia repairs, a strong fixation seemed to be the most important factor in prevention of recurrence [Figures 2 and 3]. With growing size of the mesh and true macroporous materials being used, the belief in strength reduced and gave way to the concern of acute / chronic pain possibly caused by fixation. The controversy of fixing or nonfixing the mesh is currently under scrutiny. There are reports of excellent results with meshes that are not fixed,[9,15,16] as well as some alarming ones[17,18] demonstrating an increased risk of recurrence. Since 5 years the author regularly uses bioabsorbable cyanoacrylate glue for fixation of the mesh in laparoscopic hernia repair and reserves the use of tacks or staples only for selected cases.

### Technical experience

A delicate problem for the surgical community was the issue of a new laparoscopic experience. The long learning curve of endoscopic repairs contains the potential risk of technical errors leading to unacceptable rise of recurrence rate[19] [Figure 4]. This fact highlights the need for structured well-mentored teaching, a high level of standardization of the procedure and rigorous adherence to the principles of laparoscopic hernia repair. The impact of experience on the recurrence rate was in both extremes well documented.[7–9,20] The placement of a large mesh in preperitoneal space requires wide dissection from over the midline to the proximity of the anterior iliac spine and from above of triangle of Hasselbach to below the superior arc of pubic bone and to the iliac fossa. All possible hernia orifices are checked and cord lipomas or similar formations are reduced or excluded. The wrinkle-free adaptation of the mesh on underlying tissue prevents dead spaces promoting seromas, encourages the ingrowth and reduces the risk of early dislocation.[21] In the trans-abdominal preperitoneal (TAPP) technique, meticulous peritoneal closure has to be achieved.

### Tailored approach?

Is there any form of ‘tailored approach to hernia’ today as it was postulated a decade ago? Is there an evidence for an individualized hernia repair in adult patients? From the point of view of an endoscopic preperitoneal mesh repair, the extent of dissection and the use of mesh remain the same for all types of defects. Depending on the size of the defect and ‘overall hernia risk’ factors, the choice of variable material strength, mode of fixation, form and extent may vary.

### Collagen status

Unceasing research of wound-healing processes reveals step by step the possible etiology of hernia formation. Inborn or acquired abnormalities in collagen synthesis are associated with higher incidence of hernia formation and recurrences.[22–25]

### ‘Overall risk’

The negative effect on healing in hernia repair is often related with malnutrition, obesity, steroids, type II diabetes, chronic lung disease, jaundice, radiotherapy, chemotherapy oral anticoagulants, smoking, heavy lifting, malignancy and anemia.

## CONCLUSIONS

Laparoscopic inguinal hernia repair offers excellent results in experienced hands.[8,9,15,16] The TAPP and total extraperitoneal techniques are more or less standardized. The most common causes of recurrence were recognized in the past and the necessary technical corrections were added to the best practice of laparoscopic repair. To reproduce the best quality, repair requires advanced laparoscopic skills and routine and strict adherence to the standardized principles of the preperitoneal technique. Further research on metabolic influence of tissue healing and collagen synthesis will probably markedly change our surgical practice in hernia repair and contribute to our search for excellence.
